# Care of the Patient Nearing the End of Life in the Neurointensive Care Unit

**DOI:** 10.1007/s12028-024-02064-5

**Published:** 2024-08-05

**Authors:** Hanna Ramsburg, Abigail G. Fischer, Meredith MacKenzie Greenle, Corey R. Fehnel

**Affiliations:** 1 Villanova University M. Louise Fitzpatrick College of Nursing, Villanova, PA USA; 2 Beth Israel Deaconess Medical Center, Boston, MA USA; 3 Department of Neurology, Harvard Medical School, Boston, MA USA; 4 Division of Neurocritical Care and Hospital Neurology, Department of Neurology, Beth Israel Deaconess Medical Center, Boston, MA USA; 5 Hebrew SeniorLife Marcus Institute for Aging Research, Boston, MA USA

**Keywords:** Death, Terminal care, Intensive care units, Neurology, Withholding treatment, Brain death, Bereavement

## Abstract

**Background:**

Neurologically critically ill patients present with unique disease trajectories, prognostic uncertainties, and challenges to end-of-life (EOL) care. Acute brain injuries place these patients at risk for underrecognized symptoms and unmet EOL management needs, which can negatively affect their quality of care and lead to complicated grief in surviving loved ones. To care for patients nearing the EOL in the neurointensive care unit, health care clinicians must consider neuroanatomic localization, barriers to symptom assessment and management, unique aspects of the dying process, and EOL management needs.

**Aim:**

We aim to define current best practices, barriers, and future directions for EOL care of the neurologically critically ill patient.

## Introduction

Mortality rates in US intensive care units (ICUs) approach 20%, and rates in neurointensive care units (neuro-ICUs) vary from 9 to 24%, making end-of-life (EOL) care in the ICU a common occurrence [1, 2]. Neurological diseases and injuries differ widely and yet present with unique disease trajectories, uncertain prognoses, and real challenges to symptom assessment and intervention [3, 4]. Patients in this setting have a high risk of severe physical and cognitive impairments, medical complications, and high symptom burden [4–6]. An acute brain injury often prevents patients from being able to participate in treatment decisions, placing them at risk for unmet palliative care needs that can negatively affect their quality of EOL care [4, 6].

In the neuro-ICU, a neuropalliative care approach can ensure comprehensive care with symptom management, evaluation of beliefs, documentation of values, and care and treatment preferences to ensure a comfortable death, relief of suffering, and planning for expected decline [3, 7, 8]. High-quality neuropalliative care can be delivered by the primary neuro-ICU team as well as with assistance from specialist palliative care clinicians [9]. Patients with neurological diagnoses present with unique challenges to EOL management that must be considered.

Barriers to high-quality EOL care in the neuro-ICU include prognostic uncertainty and unpredictable disease trajectory [10], altered level of consciousness or coma [11, 12], the need for surrogate decision-makers [4, 12, 13], ICU team practice variation, lack of training, misperception, and discomfort on the part of the clinician [10]. A patient’s unpredictable disease trajectory and altered level of consciousness can be challenging for surrogate decision-makers and can present barriers to ICU teams seeking reliable symptom assessment and management. Additionally, although the primary ICU team should incorporate palliative care principles into care [9], a lack of evidence specific to palliative and EOL care after severe brain injury leads to practice variation that impacts the delivery of primary palliative care. To improve clinician comfort and knowledge in providing EOL care in the neuro-ICU, we aim to describe current best practices, barriers, and future directions for EOL care of the neurologically critically ill patient. The following sections describe goals of care conversations at the EOL, potential barriers to symptom assessment and management, withdrawal of life-sustaining treatment (WLST) or transition to a comfort-only approach, and postdeath/bereavement care for families and staff.

## Goals of Care Conversations at the EOL

Having a loved one in the ICU is often traumatizing. Family members of patients admitted to the ICU are found to experience posttraumatic stress symptoms, anxiety, and depression [14]. This distress can be heightened at the EOL, with family members experiencing uncertainty secondary to variable prognoses, time pressures, difficulty of decision-making and guilt over decision-making, unidentified patient wishes, poor communication from the team, and grief related to the impending death [15–19]. Serious illness conversations between the interdisciplinary team and families may need to include an explanation of what families can expect as the patient nears the EOL. The interdisciplinary team should discuss symptom trajectory, including expected signs of the dying process (e.g., agonal respirations and upper airway sounds or “death rattle”) versus signs of discomfort or respiratory distress (airway obstruction, accessory respiratory muscle use, nasal flaring); provide education regarding pharmacological and nonpharmacological management strategies; and explain what happens after death has occurred [20].

## Potential Barriers to Symptom Assessment and Management

**Fig. 1:**
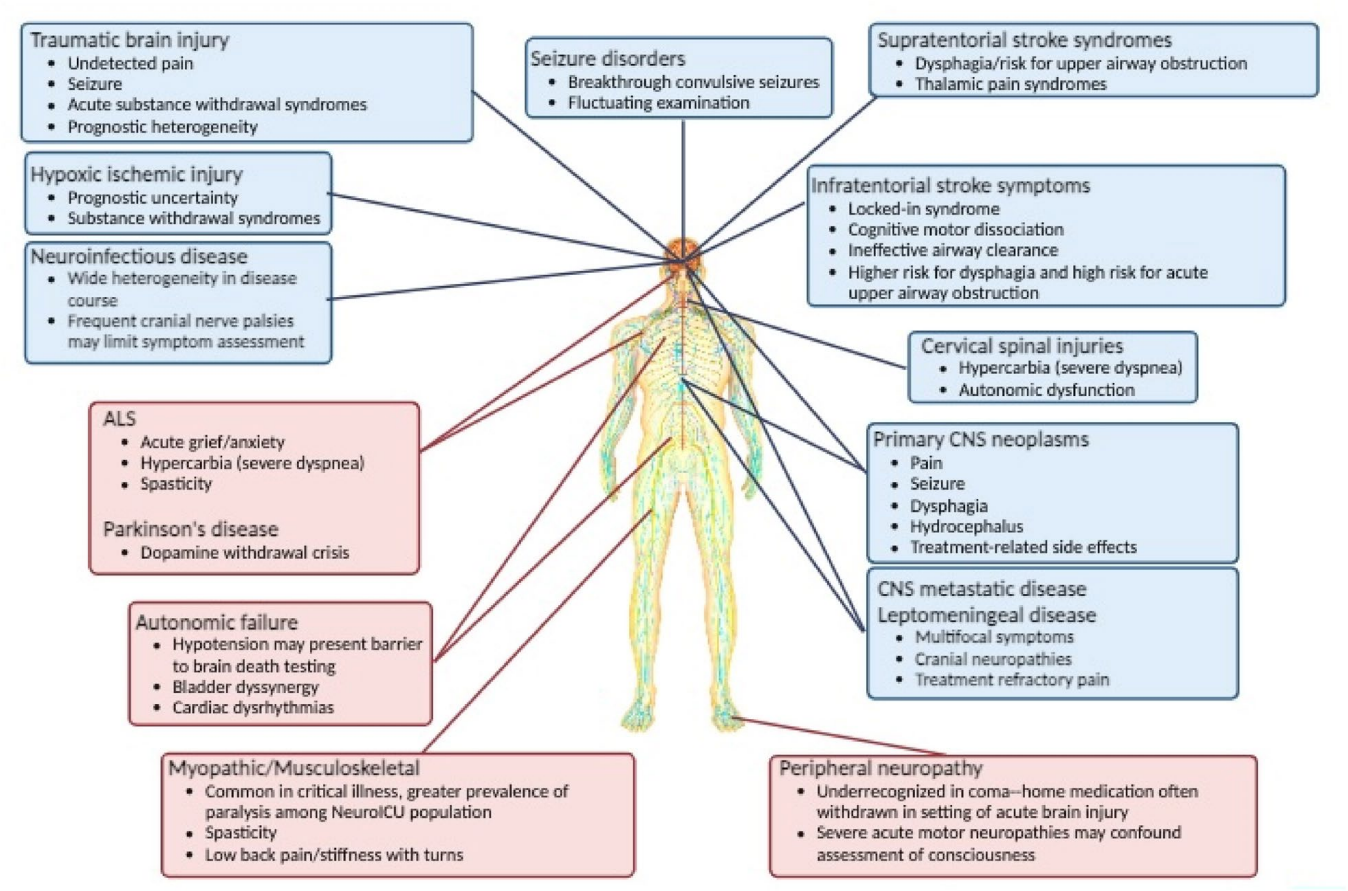
Unique considerations for end-of-life care based on neuroanatomic localization. Note. A cardinal feature of primary neurological injuries as well as states of encephalopathy is the risk for covert consciousness. Fluctuating neurological examinations are commonplace, and careful consideration should be given to this risk based on neuroanatomic localization. Blue shading indicates disorders of the CNS, and red shading indicates disorders of the peripheral nervous system. ALS amyotrophic lateral sclerosis, CNS central nervous system, NeuroICU neurological intensive care unit. Figure created with Biorender.com

Neurologically critically ill patients present unique barriers to symptom assessment and management due to the need for consideration of neuroanatomic localization. Figure 1 provides an overview of key disease states in the central and peripheral nervous systems and how each process may impact management at EOL. A cardinal feature of primary neurological injuries, as well as encephalopathy, are communication barriers and potential risk for covert consciousness (awareness not evident on bedside examination) [21]. Fluctuating neurological examinations are commonplace, and careful consideration should be given to this risk based on neuroanatomic localization (Fig. 1).

Neurologically critically ill patients may present with altered communication ability, decreased level of consciousness, motor impairment, and cognitive decline [11, 12], which can result in an underreporting of symptoms, poor symptom management, and unmet needs [11, 12, 22]. Therefore, the primary neuro-ICU care team must evaluate for alternative and often subtle signs of discomfort (e.g., tachypnea, tachycardia, agonal breathing patterns, restlessness, grimacing, diaphoresis, and accessory muscle use) [23, 24].

Table 1 addresses pharmacological and nonpharmacological options for the management of commonly occurring symptoms at EOL in neuro-ICU patients. In addition to symptoms commonly considered, EOL symptoms requiring management may also include hemiparesis, dysarthria [12], dry mouth, depression, fatigue, incontinence, and spasticity [23, 25, 26]. Therefore, to meet these patients’ EOL care needs, it is important for neuro-ICU clinicians to first identify bothersome symptoms, including neurological and nonneurological physical symptoms, psychological symptoms, and social and existential suffering [11, 27, 28]. Comprehensive symptom assessment and symptom management are key components of care for patients nearing the EOL.

**Table 1:** EOL symptoms and symptom management

Common symptoms	Symptom management [20, 85–91]
Dyspnea	• Pharmacologic ○ Opioids ▪ Morphine ▪ Hydromorphone ▪ Fentanyl ▪ Oxycodone ○ Sedatives ▪ Propofol ○ Benzodiazepines ▪ Lorazepam ○ Bronchodilators ○ Corticosteroids ○ Diuretics ○ Antibiotics • Nonpharmacologic ○ Calming environment ○ Positioning ○ Breathing techniques ▪ Pursed lip breathing ○ Oxygen ▪ Only with hypoxia ▪ Target SpO2 of ≥ 90% ○ Humidified air ○ Psychological support ○ Spiritual care
Pain	• Pharmacologic ○ Opioids ▪ Morphine ▪ Hydromorphone ▪ Fentanyl ▪ Tramadol ○ Nonopioids ▪ Acetaminophen ▪ NSAIDs ○ Adjuvant Agents ▪ Corticosteroids ▪ Anticonvulsants • Gabapentin ▪ SNRIs ▪ Local anesthetics • Lidocaine patch • Nonpharmacologic ○ Heat/cold ○ Calming environment ○ Positioning ○ sychological support
Dysphagia	• Nonpharmacologic ○ Modified food and liquid consistencies ○ Positioning ○ NPO ○ Alternative forms of nutrition and hydration
Anxiety	• Pharmacologic ○ Benzodiazepines ▪ Lorazepam ○ Antipsychotics ▪ For anxiety with agitation • Haloperidol • Nonpharmacologic ○ Listening ○ Validate emotions ○ Relaxation techniques ○ Deep breathing ○ Calming environment ○ Spiritual care
Sleep disorders (insomnia)	• Pharmacologic ○ Nonbenzodiazepine receptor modulator ▪ Zolpidem ○ Tetracyclic antidepressant ▪ Mirtazapine ○ SARIs ▪ Trazodone ○ Benzodiazepines ▪ Lorazepam • Nonpharmacologic ○ Sleep hygiene ○ Relaxation techniques
Seizures	• Pharmacologic ○ Anticonvulsants ▪ Phenobarbital ▪ Phenytoin ▪ Valproate ○ Benzodiazepines ▪ Lorazepam
Hypercarbia	• Pharmacologic ○ Bronchodilators ○ Sodium bicarbonate ○ Opioids ○ Benzodiazepines • Nonpharmacologic management ○ Positioning ○ Calming environment ○ Bilevel positive airway pressure ▪ If consistent with patient’s goals
Delirium/agitation/restlessness	• Pharmacologic ○ Antipsychotic ▪ Haloperidol ▪ Olanzapine ▪ Risperidone ○ Sedatives ▪ Propofol ○ Benzodiazepines • Nonpharmacologic ○ Calming environment ○ Promote sleep/wake cycle ○ Caregiver at bedside
Nausea/vomiting	• Pharmacologic ○ 5-HT3 serotonin-receptor antagonist ▪ Ondansetron ○ Benzodiazepines ▪ Lorazepam ○ Dopamine receptor antagonists ▪ Metoclopramide ○ Phenothiazines ▪ Promethazine ▪ Prochlorperazine ○ Corticosteroids ▪ Dexamethasone
Excessive secretions	• Pharmacologic ○ Anticholinergics ▪ Atropine ▪ Scopolamine ▪ Glycopyrrolate ▪ Hyoscyamine • Nonpharmacologic ○ Oral suction ▪ Only for comfort ○ Positioning ○ Discontinue fluids ○ Calming environment
Constipation	• Pharmacologic ○ Laxative ▪ Senna ▪ Bisacodyl suppository ○ Stool softener ▪ Docusate

EOL, end of life, NPO, nothing by mouth, NSAID, Non-steroidal anti-inflammatory drugs, SARIs, serotonin receptor antagonists and reuptake inhibitors, SNRIs, Serotonin–norepinephrine reuptake inhibitors, SpO2, Oxygen saturation

The second barrier to symptom assessment and symptom management is the lack of a standardized neurological EOL assessment tool. In 2017, a systematic review identified 152 palliative care assessment tools that cover structure and process, physical, psychosocial, social, existential, cultural, ethical and legal, and multidimensional domains of care [29]. Despite the abundance of palliative care assessment tools, a majority of comprehensive assessment tools have almost exclusively been evaluated in patients with cancer, focused mainly on physical care [30], or were validated in medical/surgical/cardiac ICUs, leading to a wide variability in symptom identification.

**Fig. 2:**
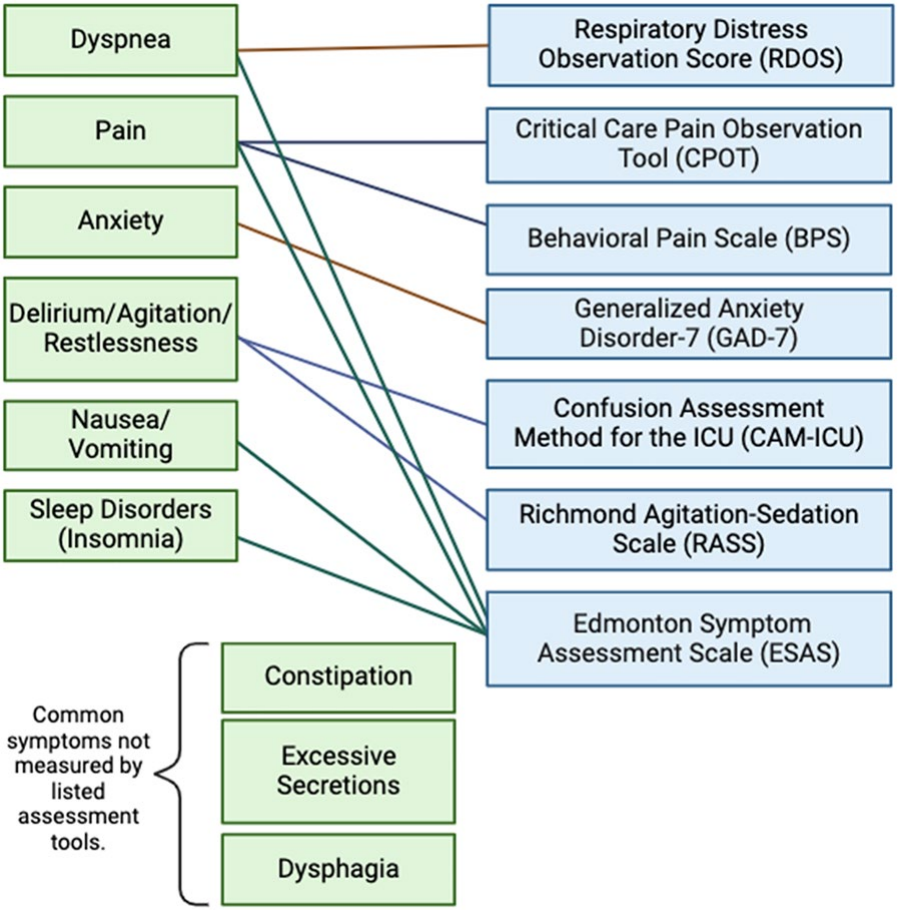
Tools for symptom assessment. Note. Assessment tools are arranged from single-item to multi-item symptom assessment tools. Figure created with Biorender.com

To help identify neuro-ICU patients’ neuropalliative care needs, clinicians can use checklists and screening tools. Checklists such as the SuPPOrTT checklist offer a timely assessment of palliative care needs [6]. In comparison, screening tools may vary in length and time needed for completion. Figure 2 shows commonly used single-item and multi-item symptom assessment tools, including the Respiratory Distress Observation Scale [31], the Critical Care Pain Observation Tool [32, 33], the Behavioral Pain Scale [33, 34], the Generalized Anxiety Disorder-7 (GAD-7) [35], the Confusion Assessment Method for the ICU [36], the Richmond Agitation Sedation Scale [37], and the Edmonton Symptom Assessment Scale [38]. Of note, the GAD-7 [35] and the ESAS [38] require patient cooperation and may not be appropriate for all neuro-ICU patients. However, these tools offer a quick scaled assessment of distressing symptoms and can be easily incorporated into neuro-ICU symptom assessments.

Notably, many palliative care assessment tools used in neurological diseases have been adapted from existing tools that were developed for nonneurological diseases. Further, validation studies of many clinical symptom rating scales excluded patients with severe neurological disease, who are commonly cared for at EOL in the neuro-ICU setting. Therefore, the careful selection of multiple tools targeting distressing symptoms specific to the neurological injury [28] is often needed. Because of continued gaps in symptom assessment, validation of existing tools as well as the development of new ones specific to neurological disease is needed.

The use of screening tools, checklists, questionnaires, and scales may promote systematic screening and management of neuropalliative symptoms and allow for referrals to palliative care specialists for complex refractory symptoms if needed [13]. The neuro-ICU captures a unique group of patients with requirements for screening tools and assessments tailored to the nature of the neurological injury. Although further investigation will support scale development and validation, establishing best practices for implementation and measures of success for these processes is needed.

## Coma and Risk for Covert Consciousness

Coma is a hallmark of acute brain injury requiring neurocritical care and is a key barrier to symptom assessment and management at EOL. Heterogeneity among structural and metabolic etiologies for coma among neurologically critically ill patients can lead to a risk for undetected or “covert” consciousness. In the setting of clinical coma, bedside examination is limited by restriction to cranial nerves and motor responses that do not fully interrogate the extent of nervous system function. Further, early investigations have suggested that validated detailed examination techniques, such as the Coma Recovery Score, may fail to identify consciousness in as many as 20% of patients [39]. Although the preservation of awareness at EOL among neurologically critically ill patients is likely lower than in studies of patients surviving their brain injury, a report found that task-based functional magnetic resonance imaging identified a patient transitioning to comfort-focused care at EOL with covert consciousness [40]. Clinicians caring for the patients at EOL in the neuro-ICU should not be assured that a bedside examination supporting coma is an accurate reflection of the patient’s experience. This limitation is crucial in developing a structured plan for EOL symptom assessment, analgesia, and sedation.

A careful neuroanatomic and toxic-metabolic evaluation for coma must be considered in planning EOL care. Figure 1 outlines major conditions, their neuroanatomic regions of interest, and their potential implication for symptom management at EOL. A few examples to consider include the following: Does a patient with basilar artery thrombosis, initially felt to be “locked-in,” who loses the ability to communicate after developing pneumonia have the potential for retained consciousness? or What is the symptom experience of patients with diffuse axonal injury in traumatic brain injury or bilateral frontal injuries? An abundance of caution must be used in determining lack of consciousness based on traditional bedside examinations. We recommend open discussion of the possibility for covert consciousness with a patient’s surrogate decision-maker. This includes sharing the plan for provision of sedation and analgesia, even in situations in which consciousness may not be probable. Depending on the clinical situation, the palliative use of sedation at EOL can be a complex decision-making process; however, recent guidelines outlining its indication for use in WLST can provide a useful framework [41].

## Comfort-Focused Transitions and the Dying Process Among Neuro-ICU Patients

The process of transitioning to comfort-focused care in the neuro-ICU requires unique consideration because of the prevalence of coma. Although peripheral nervous system diseases also present challenges to assessment and comfort (Fig. 1), they are either secondary to critical illness itself or more readily responsive to treatment (e.g., myasthenic crisis or acute inflammatory demyelinating polyneuropathy). Although the endotracheal tube is a barrier to verbal communication among patients with peripheral nervous system disorders, central nervous system (CNS) dysfunction compounds limitations to symptom assessment.

Most people who die in ICUs do so after withholding life-sustaining treatment or WLST. WLST in the neuro-ICU can include mechanical ventilation, artificial nutrition and hydration, vasopressor support, hemodialysis, extracorporeal membrane oxygenation (ECMO), and sometimes antibiotics and other medications. Multiorgan failure is common in critical illness, but heart and lung dysfunction is frequently of lesser severity relative to the primary neurological injury in neuro-ICUs. The most common means of WLST in the ICU setting, and particularly the neuro-ICU, is palliative withdrawal of mechanical ventilation (WMV) [42]. Isolated CNS injury in the absence of prominent cardiovascular or pulmonary injury limits our understanding of the expected course once WMV occurs.

## Palliative WMV in the Neuro-ICU

Nearly one in five people in the United States will die either in or shortly after ICU care [43, 44]. The most common process for WLST in the ICU setting is the palliative WMV. Despite the high frequency of the event, evidence guiding the care of patients at EOL in the ICU is limited [45]. Although critical illness severity scores reliably predict mortality, risk factors for predicting distress at EOL in the ICU are not well established. Uncertainty of the adequacy of airway protective reflexes in the setting of acute brain injury and secondary lung dysfunction acquired during critical illness (e.g., aspiration pneumonia, volume overload, pulmonary embolism) can make WMV in the neuro-ICU particularly challenging.

Across all critically ill patients, there is growing evidence of frequent distress among patients undergoing WMV at the EOL [42, 46]. Although neurologically critically ill patients have lower rates of primary lung disease and survey data suggest patients experience less distress [47], objective estimates of rates of severe tachypnea approach 19–30% [46].

Approaches to palliative WMV vary, and the optimal approach specific to neurocritical care patients remains unclear. The largest observational study across ICUs in France compared immediate extubation with terminal weaning (stepwise reduction in ventilator support) [48]. Immediate extubation was associated with a greater incidence of gasping and obstruction, whereas terminal weaning resulted in modestly higher job strain [49] among clinicians. Limited evidence suggests that extubation and family presence at the time of death may be associated with better family satisfaction [50]. A recently completed randomized controlled trial comparing usual care to a nurse-driven algorithm for WMV using the Respiratory Distress Observation Score revealed lower rates of distress in the intervention arm [31, 42].

Patients in the neuro-ICU require symptom management approaches tailored to their site of neurological injury at EOL (Fig. 1). Many procedures at EOL (e.g., extubation) can be expected to result in distress. Although pain is an important symptom to be identified and treated, a growing body of evidence suggests dyspnea or respiratory distress is not only more common but also far more distressing and often goes unrecognized [51]. There is high-quality evidence for opiates as most effective for alleviating dyspnea [52, 53]. However, during WMV at EOL, it is not known whether administering analgesia/sedation prior to extubation (anticipatory dosing) relieves distress more effectively than giving these drugs only in response to observed symptoms (reactive dosing) [54, 55]. Most published studies suggest anticipatory dosing is not associated with earlier time to death [46, 56, 57]. There is limited high-quality evidence around sedation and other pharmacological practice at EOL. Consideration of known preservation of consciousness or the potential for covert consciousness should be paramount in decision-making regarding the need for sedation. If needed, sedatives with a narrow therapeutic window of effect (e.g., propofol) may be exchanged for others less likely to suppress respiratory drive (benzodiazepines).

## EOL Management Unique to the Neuro-ICU

Refractory cerebral edema and raised intracranial pressure are often managed with use of invasive monitoring and treatment devices, which can pose a challenge to EOL care. Once care is transitioned to a comfort focus, extraventricular drains are often closed; however, it is variable as to whether the devices are removed. Similarly, there is variation as to whether other invasive intracranial monitoring devices remain in place or are removed at the time of transition to comfort-focused care. At present, there are no known studies of family perspectives on this practice; therefore, the patient’s family/surrogate should be engaged about removal of intracranial monitoring devices. Given the heterogeneity of coma, appropriate use of analgesia should be used for painful portions of device removal (e.g., staples or sutures). Hyperosmolar therapy is generally discontinued because it may artificially prolong the dying process and risk for suffering.

Although most acute CNS processes are associated with an increased seizure risk, patients known to have seizures or refractory status epilepticus present a particular challenge for symptom management at the EOL [58]. Breakthrough seizures are not only detrimental to the patient but also commonly distressing to families and care teams. Among patients with postanoxic myoclonic status epilepticus, continuous video electroencephalogram (EEG) can be helpful in differentiating myoclonic seizures from postanoxic myoclonus without an EEG correlate. Once the seizure phenotype has been characterized, EEG monitoring may be discontinued in preparation for the transition to comfort-focused care. Decision-making for antiseizure drug management should be based on the patient’s disposition plan. If the patient will remain hospitalized for EOL care, antiseizure drugs should be continued. However, if enteral access is not to be maintained or transfer to a hospice facility or home is planned, benzodiazepines remain the mainstay of antiseizure therapy at EOL. Careful dose conversion of antiseizure drugs to a standing benzodiazepine regimen or continuous infusion may be warranted. The frequency of dosing of most antiseizure drugs may constrain medication conversion in this setting, and phenobarbital or ketamine may be considered [59].

Dysphagia and a lack of enteral access can result in a myriad of worsened symptoms among patients with chronic neurological diseases who may be cared for in the neurocritical care unit at EOL. Separate from epilepsy, refractory symptoms among patients with movement disorders can be common at EOL. Abrupt withdrawal of dopaminergic agents among patients with Parkinson disease may result in parkinsonism hyperpyrexia syndrome [60]. Rotigotine patches can be helpful when enteral access is not possible; however, onset is delayed and necessitates liberal use of benzodiazepines and opiates when necessary [61].

## Care of the Potential Organ Donor

### Organ Donation After Circulatory Determination of Death and Death by Neurological Criteria (Brain Death)

Organ donation after the circulatory determination of death (DCDD) presents a unique set of challenges, particularly for the neurologically critically ill. Experienced multidisciplinary teams may be best suited for the complex decision-making and unique environment of DCDD [62]. Both DCDD and death by neurological criteria have wide-ranging impacts that deserve in-depth descriptions that are beyond the scope of this article [63]. Careful adherence to current recommendations for establishing the diagnosis of death by neurological criteria can alleviate any concerns for misdiagnosis and therefore potential for distress [62, 64].

## Postdeath Care/Bereavement Care

Although multiple frameworks and models exist for the provision of postdeath care, there are seven main elements identified in the literature. These are as follows: (1) notification of survivors, (2) organ donation, (3) completion of the death certificate, (4) postmortem care with transition from the bedside to the morgue, (5) delivery of detail for next steps to family/friends, (6) bereavement care, and (7) health care team debrief [65–67].

### Bereavement Care for Family Members

Caregivers, family members, and friends of patients who die in the ICU are at risk for developing complicated grief, emotional distress [68, 69], and post-intensive care syndrome family (consisting of depression, anxiety, acute stress disorder, complicated grief, and posttraumatic stress disorder) [70]. Factors that could lead to these poor bereavement outcomes in the ICU include the following: poor communication, lack of involvement in decision-making, absence at time of death, perception of inadequate symptom management and poor EOL care, and feeling unsupported [68, 70, 71].

Bereavement is a unique experience that requires individual assessment [72] and should include support services before and after death [65–67, 73]. Quality bereavement care should include clear communication, EOL discussions, and information for additional supportive resources [65–67, 69, 71, 74]. Bereavement information should include what to expect during the dying process, frequent patient updates in easy-to-understand language, what happens after death, what to expect in the grieving process, where to access bereavement support, and details for additional resources [65–67, 70]. Notably, research suggests that bereavement information should use a combination of different formats of information. For example, verbal communication and written information in the form of a phone call, brochure, and/or condolence card [70, 74].

Although bereavement care should be a standard and routine part of clinical practice in the ICU, challenges to bereavement care may include balancing bereavement care with clinical workload, lack of bereavement education, and limitations of the hospital environment [75, 76]. To best support the provision of bereavement care, ICU health care clinicians need adequate education and training opportunities, protected clinical time, a private space to provide care, and use of an interdisciplinary team approach [65, 70, 73].

### Bereavement Care for Health Care Clinicians

Health care clinicians in the ICU frequently encounter poor patient outcomes and death, which puts them at risk for psychological distress [71, 77, 78], such as unresolved feelings of grief [75, 77], burnout [77–79], and compassion fatigue [75, 79]. Therefore, bereavement care for health care clinicians in the ICU is essential to ensure the continued provision of high-quality EOL care.

Bereavement care for clinicians can consist of a combination of self-care strategies [80] and debriefing sessions [81, 82]. Self-care strategies may include the following: finding meaning in work, connecting with an energy source (spiritual beliefs, family support, or social connections), developing a positive attitude, nurturing personal connections, recognizing one’s uniqueness, and performing emotional hygiene (self-reflecting, setting boundaries, spending time with family and friends, exercising, getting adequate sleep, and praying) [80]. A team debriefing session may involve reflection of the hospital course, a moment of silence, identification of what went well, discussion of concerns or questions, and identification of things for which the team is grateful [67]. Providing bereavement care among health care clinicians can lead to effective coping [75, 81], improve connection among team members [75, 81], reduce burnout [80], and support the provision of high-quality EOL care.

## Conclusions and Implications for Future Research

Neuropalliative care challenges for patients nearing the EOL in the neuro-ICU include variable disease courses, prognostic uncertainty, high symptom burden [3, 12], gaps in training [13], and EOL management consideration unique to the neuro-ICU. Thus, neuropalliative care principles should be proactively incorporated into care at the time of admission to the neuro-ICU [13]. These include goals of care conversations, neuroanatomic considerations, symptom assessment and management, planning for expected decline, and support for the patient, family, and health care provider [11–13, 83].

Future research in the neurologically critically ill population is needed to address these challenges and can be accomplished through assessment tool development and EOL educational training. First, to better identify specific neuropalliative care needs in the neuro-ICU, the development and validation of a comprehensive assessment tool for EOL symptoms specific to neurological diseases while encompassing symptoms shared by all critically ill patients is needed [22]. Second, all neuro-ICU health care clinicians should be properly trained in EOL symptom assessment, management, and bereavement care [84]. Training programs such as the End-of-Life Nursing Education Consortium and Center to Advance Palliative Care may be beneficial to support the provision of high-quality palliative and EOL care in all practice settings. Lastly, training programs must be informed by high-quality evidence supporting the effectiveness of interventions designed to reduce distress at EOL experienced by patients, their families, and the ICU teams caring for them.
